# Detection and characterization of two co-infection variant strains of avian orthoreovirus (ARV) in young layer chickens using next-generation sequencing (NGS)

**DOI:** 10.1038/srep24519

**Published:** 2016-04-19

**Authors:** Yi Tang, Lin Lin, Aswathy Sebastian, Huaguang Lu

**Affiliations:** 1 Wiley Lab/Avian Virology, Animal Diagnostic Laboratory, Department of Veterinary and Biomedical Sciences, The Pennsylvania State University, University Park, PA 16802, United States; 2 Department of Biochemistry and Molecular Biology, The Pennsylvania State University, University Park, PA 16802, United States

## Abstract

Using next-generation sequencing (NGS) for full genomic characterization studies of the newly emerging avian orthoreovirus (ARV) field strains isolated in Pennsylvania poultry, we identified two co-infection ARV variant strains from one ARV isolate obtained from ARV-affected young layer chickens. The de novo assembly of the ARV reads generated 19 contigs of two different ARV variant strains according to 10 genome segments of each ARV strain. The two variants had the same M2 segment. The complete genomes of each of the two variant strains were 23,493 bp in length, and 10 dsRNA segments ranged from 1192 bp (S4) to 3958 bp (L1), encoding 12 viral proteins. Sequence comparison of nucleotide (nt) and amino acid (aa) sequences of all 10 genome segments revealed 58.1–100% and 51.4–100% aa identity between the two variant strains, and 54.3–89.4% and 49.5–98.1% aa identity between the two variants and classic vaccine strains. Phylogenetic analysis revealed a moderate to significant nt sequence divergence between the two variant and ARV reference strains. These findings have demonstrated the first naturally occurring co-infection of two ARV variants in commercial young layer chickens, providing scientific evidence that multiple ARV strains can be simultaneously present in one host species of chickens.

Avian orthoreovirus (ARV), the *Orthoreovirus* genus in the *Reoviridae* family^1^,^2^, is a highly contagious avian species virus. The ARV virion averages 70–80 nm in size, containing a non-enveloped icosahedral double-layered capsid structure and 10 double-stranded (ds) RNA genome segments^3^. The segmented genomic dsRNAs are divided into 3 different groups, including large segments (L1, L2, and L3), medium segments (M1, M2 and M3), and small segments (S1, S2, S3 and S4), according to mobility in polyacrylamide gel electrophoresis (PAGE)^4^,^5^. Together, these genome segments encode at least 8 structural proteins (λA, λB, λC, μA, μB, σA, σB and σC) and 4 nonstructural proteins (μNS, p10, p17 and σNS). The structural proteins are incorporated into progeny ARVs, while the nonstructural proteins are encoded within the viral genome but are not observed in the mature ARV virion^6^. The first seven bases (5′-GCUUUUU-3′) at the 5′ untranslated regions (UTRs) and the last five bases (5′-UCAUC-3′) at the 3′ UTRs of each ARV genome segment are highly conserved in known ARV strains^7^.

ARVs are important pathogens of domestic poultry and wild avian species, causing a variety of clinical diseases, with viral arthritis/tenosynovitis as the primary infection^2^,^8^. ARV-associated viral arthritis is an important disease problem in meat-type chickens, turkeys^9^ and layer chickens^10^. Newly emerging ARV variants or novel ARV strains have occurred, causing severe lameness and arthritis diseases in Pennsylvania (PA) poultry since 2011 until the present^9^,^11^. The genetic diversity and reassortment between the newly emerging ARV variants and classic vaccine strains have resulted in vaccine failure in breeder flocks with routine ARV immunity programs^12^.

As a segmented genome virus, ARV genes can reassort to introduce drastic changing in the genotype and pathotype through a direct exchange of genome segments^13^,^14^. Co-infections with genetically distinct ARV strains can lead to the generation of recombinant viruses, confirmed through experimental co-infections of chicken embryonic fibroblast cells with two ARV strains^15^. However, the diagnosis of two or multiple ARV strain co-infections is difficult in field cases, reflecting the limitations of physically isolating genome segments and high-frequency reassortment in some ARV segments^14^. Indeed, until recently, field cases of ARV co-infections have not been reported.

Next-generation sequencing (NGS) is a high-throughput sequencing methodology that generates millions of sequencing reads^16^,^17^ and sequences directly from viral RNA. NGS has made revolutionary changes and offered new perspectives in complete genome sequencing studies^18^. In the present study, we described two co-infection ARV variant strains detected in one ARV field isolate obtained from ARV-affected young layer chickens in PA. These research findings provide scientific data for the confirmation of the naturally occurring co-infection of two ARV variant strains in layer chickens.

## Results

### ARV isolate and Sanger sequencing

The ARV isolate (Reo/PA/Layer/01224/14) conducted in the present study was isolated from the tendon tissues of ARV-affected young layer chickens in PA in 2014^19^. The isolate was propagated in LMH (ATCC CRL-2117) hepatocellular carcinoma cell cultures. The viral RNA was amplified at 1088 bp using σC-based one-step RT-PCR with P1/P4 primers^20^ from both original tendon tissue sample and ARV positive cell culture supernatant. Direct Sanger sequencing for the σC gene RT-PCR products form original tendon tissue sample and ARV positive cell culture supernatant identified one σC gene sequence of ARV isolate(s) (KP727789) exhibited approximately 90% nt identity with an ARV strain in GenBank (KC865792). The RT-PCR-positive viral RNA was processed for NGS.

### NGS data analysis

**Figure 1:**
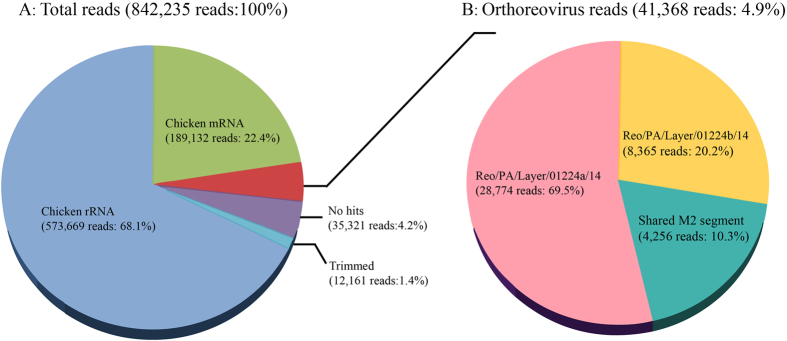
Pie chart illustrating the homology search results for NGS reads. (**A**) Total NGS reads homology search results; (**B**) Orthoreovirus NGS reads homology search results.

From the total RNA sample of the layer ARV isolate after propagation in LMH cell cultures, a total of 842,235 sequencing reads were generated, resulting in 251 Mb of fastq format sequence data. After default quality control (QC) filter processing on the MiSeq platform, the sequencing reads were aligned to the reference sequences of chicken genomic DNA and rRNA databases, followed by quality trimming to remove low-quality reads and to exclude reads with similarities to chicken mRNA or rRNA sequences. As a result, 573,669 reads (68.1%) were identified as chicken rRNA, 189,132 reads (22.4%) as chicken mRNA, and 12,161 reads (1.4%) as sequencing adapters (Fig. 1A). The remaining 76,689 reads (9.1%) were considered clean reads and were further analyzed using BLASTN, revealing 35,321 reads (4.2%) as no hits and 41,368 reads (4.9%) as orthoreovirus-origin (Fig. 1B).

### *De novo* assembly of viral genomes

**Figure 2:**
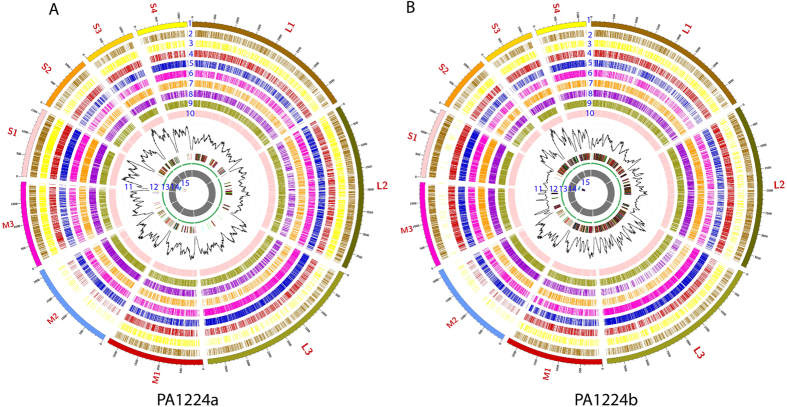
Circos plot descriptions of the complete genomes of the two co-infection variant strains. (**A**) Reo/PA/Layer/01224a/14 variant (or PA01224a); (**B**) Reo/PA/Layer/01224b/14 variant (or PA01224b); Track 1: Consensus sequence; Tracks 2–10: Sequence variations of the two variants of PA01224a and/or P101224b compared with PA15511, PA05682, S1133, 1733, 138, AVS-B, MN9 and J18, respectively; Track 11: Sequencing depth of NGS, the axis of the coverage track corresponds to 0, 100, 200, 300, 400, and 500 reads from inside to outside; Track 12: Assembled contigs using de novo assembly (SPAdes); Track 13: Open reading frames (ORFs); Track 14: σC gene Sanger sequencing results; Track 15: Sequence variations between NGS and Sanger sequencing results in the σC gene.

After *de novo* assembly using the SPAdes program, the clean reads generated a total of 52 contigs, varying from 109 to 3942 nt in length. After BLASTN searching of the 52 contigs, 19 contigs (Table 1) were identified to be ARV sequences, and 9 of the 10 ARV genome segments were targeted by two homologous contigs, except M2 targeted by one contig. These findings indicated there were two genomes of ARV variants with 9 different genome segments and one same M2 segment in the sequencing sample (Table 1). The sequencing depth at every base of the contigs was shown on track 11 of the circos plots (Fig. 2A,B). Highest similarity searching of the 19 ARV-related contigs in GenBank revealed that all 19 contigs had different homologies with other published reference ARV strains (82–98%). To obtain the sequencing coverage data, the sequencing reads were mapped back to the assembled contigs. The reads coverage was calculated from 26.82× to 254.01× on average for each segment. The mapped reads of each segment varied from 404 to 5978 reads, which positively correlated with the sequencing coverage data (Table 1). To identify the intra-host single-nucleotide variants (iSNVs) in the assembled contigs, the reads mapping results were processed using the resequencing program of CLC Genomics Workbench software with a 0.4% sequencing error correction. A total of 21 iSNVs were determined in five contigs corresponding to L1, L3, M1, S2, S3 and S4 segments (Table 1), and 17 of the 21 iSNVs had sequencing depths greater than 100×.

### Separation of the viral genome through sequencing coverage

NGS deep sequencing revealed two ARV genomes in the prepared total RNA sample, indicating that this layer chicken ARV isolate contained two variant strains or that the ARV-affected layer chickens were co-infected with two variant strains of ARV. After assessing the sequencing coverage data, two contigs of each ARV segment were separated based on high and low sequencing depths into two groups, except for the M2 segment. The contigs in the high-coverage group were designated as one ARV strain (Reo/PA/Layer/01224a/14, or PA01224a), whereas the contigs in the low-coverage group were designated as the other strain (Reo/PA/Layer/01224b/14, or PA01224b) (Table 2). The statistical significance between the coverage of the two groups was confirmed using Student’s t-test (p < 0.01), indicating the reliability of the separation of viral genome. After the 41,368 orthoreovirus reads were mapped back to the PA01224a and PA01224b genomes, 28,774 reads (69.5%) were identified as the PA01224a genome, 8,365 reads (20.2%) as the PA01224b genome, and 4,256 reads (10.3%) as the M2 segment (Fig. 1B).

### The complete genomes of the two co-infection variant strains

The complete genomes of PA01224a and PA01224b were obtained after adding the same M2 segment to each contigs group. The full-length sequences of the two co-infection variant strains have been deposited into GenBank (PA01224a, KT428298 to KT428307; PA01224b, KT428308 to KT428317). The complete genomes of both strains were each 23,493 bp in length, with an approximately 50% G + C content and 10 dsRNA segments encoding 12 viral proteins. The lengths of the genomic segments of the two co-infection variant strains ranged from 1192 bp (S4) to 3958 bp (L1), and the sizes of the open reading frames (ORFs) ranged from 3882 bp (λA) to 300 bp (p10), similar to classic ARV strains. The ORF prediction of each segment indicated the existence of one tricistronic segment (S1) and nine monocistronic segments. Even the sizes of putative proteins, encoded by nine monocistronic segments, were identical between the two co-infection variant PA01224a and PA01224b strains, but the non-structural p17 gene on the S1 segment showed some differences. The p17 gene of PA01224a was 459 nt (153 aa) in length, whereas PA01224b has a smaller p17 gene, with 441 nt (147 aa) (Table 2). The UTRs were located at the 5′ and 3′ ends of each segment, ranging from 12–30 bp (5′ UTRs) and 33–98 bp (3′ UTRs) in length, respectively (Table 2). The highly conserved regions in the 5′ UTR (5′-GCUUUU-3′) and 3′ UTR (5′-UCAUC-3′) were also detected in all of the segments of the two co-infection variant strains (Table 3).

### Additional experiments for validation of the two co-infection variant strains in the original tendon sample

The σC gene RT-PCR product amplified at 1088 bp from the original tendon tissue sample was inserted into the pGEM-T easy vector for cloning improvement of Sanger sequencing. A total of 14 successful colonies were obtained, and their recombinant plasmids were processed for Sanger sequencing. We identified 12 of the 14 colonies containing the identical σC gene sequence (KP727789) as obtained in direct Sanger sequencing from the ARV cell culture sample, and the remaining 2 colonies contained the heterogeneous σC gene (KU726094) exhibited approximately 98% nt identity with a reference ARV strain in GenBank (KP731617) but only 58% nt identity with the other 12 colonies’ σC gene (KP727789). Unfortunately, the extracted RNA from the original tendon tissue could not be processed for NGS because the RNA concentration was too low to pass the quality control step of NGS.

### Sequence comparison

The nt and aa sequences of the two co-infection variant strains were initially compared, followed by a comparison with eight reference ARV strains, including two recently published PA broiler ARV strain (Reo/PA/Broiler/05682/12, or PA05682 and Reo/PA/Broiler/15511/13 or PA15511)^11^,^21^, four reference chicken-origin ARV strains (S1133, 1733, 138, and AVS-B), one turkey ARV strain (MN9), and one duck ARV strain (J18) (supplementary Table S1). The results are summarized in Table 4 and illustrated in tracks 2–10 of Fig. 2A,B. The nt and aa sequence comparisons between the two co-infection variant strains of PA1224a and PA1224b revealed low to high similarities (nt: 58.1–100%; aa: 51.4–100%). In addition to the same M2 segment coding μB gene, the highest sequence similarity was observed not only in the σA genes (nt: 88.8%; aa: 97.8%) of the two strains, but also in the λA, λC, μA, σB, and σNS genes (nt: >87%, aa: >95%). The lowest similarity was observed in the σC gene on the S1 segment, sharing only 58.1% nt identity and 51.4% aa identity. When compared with ARV reference strains, the PA01224a variant strain showed high identity with ARV strain 138 in λA- and μA-encoding genes (nt: 90.3–90.7%; aa: 97.3–98.4%); 1733 in μNS-, σA-, and σB-encoding genes (nt: 88.9–91.8%; aa: 94.3–97.1%); AVS-B in λB- and σNS-encoding genes (nt: 90.8–92.7%; aa: 98.3–98.4%) and PA15511 in λC-encoding genes (nt: 94.5%; aa: 97.7%). For the PA01224b variant strain, the sequence comparison results also indicated high identity between this study strain and chicken-origin ARV strains. The highest identities were observed in λA-, μNS-, σA-, and σB-encoding genes of the 1733 strain (nt: 81.3–91.3%; aa: 92.0–98.3%), in λC-, μA−, σNS-encoding genes of AVS-B strain (nt: 89.9–95.0%; aa: 97.1–98.4%) and in λB- and σC-encoding genes of PA15511 strain (nt:93.7–98.4%; aa: 98.8–99.0%). The results of the nt and aa sequence comparisons of σC-encoding genes revealed low identities between the two co-infection variant strains and reference strains (nt: ≤65.3%; aa: ≤60.2%). In contrast, the two variant strains of PA01224a and PA01224b exhibited the highest similarities (nt: 97.1%; aa: 98.7%) in the same μB-encoding genes with the PA broiler strain PA05682. Although isolated from different species, turkey ARV-MN9 showed moderate to high identity (nt: 75.1–87.1%; aa: 89.2–96.8%) with the two co-infection variant strains, except for σC-encoding genes (nt: 53.3–56.3%; aa: 48.3–51.0%). Duck-origin ARV-J18 was confirmed as the most divergent strain (nt: 40.4–78.1%; aa: 30.3–95.1%) from the two ARV co-infection variant strains among all reference ARV strains in sequence comparisons.

Comparison of the UTRs revealed that the genome segments of all of the ARV strains shared a common motif in the 5′ and 3′ UTRs. The seventh base of the 5′ UTR was conserved in strains S1133, 2408, MN9 and J18 but showed heterogeneity in other compared strains, including PA01224a and PA01224b. In the 3′ UTR, the 5′-UAUUCAUC-3′ motif was shared by all ARVs, although the second uracil might be replaced with cytosine in some segments (Table 3).

### Phylogenetic analysis of the two co-infection variant strains

**Figure 3:**
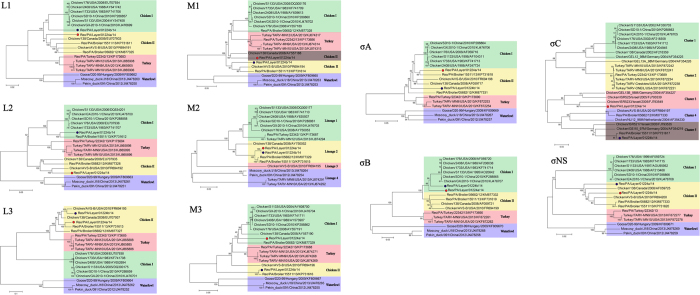
Phylogenetic trees constructed with avian orthoreoviruses (ARVs) based on nucleotide sequences of the L-, M- and σ-class homologous genome segments or genes. Note: The two co-infection variant strains are indicated with colored dots: red indicates Reo/PA/Layer/01224a/14, and blue indicates Reo/PA/Layer/01224b/14.

To examine the evolutionary relationships of the two co-infection variant strains with other ARV members, both variant strains of PA01224a and PA01224b were subjected to phylogenetic-tree analysis (Fig. 3). Rooted maximum likelihood phylogenetic trees were generated based on the nt sequence alignments of six genome segments and four σ-class genes. For the L-class segment analysis, the two co-infection variant and reference strains formed 4 host-related groups in all three segments (L1–L3), of which two groups were chicken-origin, and the other two variants were, respectively turkey- and waterfowl-origin. The two variant strains of PA01224a and PA01224b were divided into different chicken-origin ARV groups in L1 and L2 segments (Fig. 3, L1 and L2). Although the two co-infection variant strains were in the same group in the L3 tree, strain PA01224b showed a closer evolutionary relationship with AVS-B and 138 strains compared with PA01224a (Fig. 3, L3). For the M-class segments analysis, host-related groups were also observed in M1 and M3 trees, and the two co-infection variant strains evolved into separated groups in these two segments (Fig. 3, M1 and M3). Four genotyping lineages were formed through different combinations of ARVs of different species origins in the M2 tree. The same M2 segment of the two co-infection variant strains belonged to lineage 2, together with other two PA ARV field strains (PA05682 and PA15511)^11^,^21^ and one classic ARV strain 138 (Fig. 3). The phylogenetic trees of σ-class genes illustrated a close relationship between the two co-infection variant strains of PA01224a and PA01224b, particularly in the σA, σB and σNS genes (Fig. 3). Genes σA and σNS evolved distantly from all vaccine strains and formed a separated genotyping group with the pathogenic ARV strains. In contrast, the σB genes of the two co-infection variant strains were closely related with the vaccine strains, suggesting the possibility of the reassortment of S3 segments between the co-infection variant strains and vaccine strains. Gene σC was the most diverse among all 10 ARV segment genes, and the construction of a σC phylogenetic tree using co-infection variant strains and reference strains generated five genotyping clusters, showing more than 70% identity within each cluster. The two co-infection variant strains were grouped into genotyping cluster 3 (PA01224a) and cluster 5 (PA01224b), respectively, exhibiting markedly higher divergence with vaccine strains than with other segments, as indicated in the sequence comparisons.

### Visualization of the whole genome alignment

**Figure 4:**
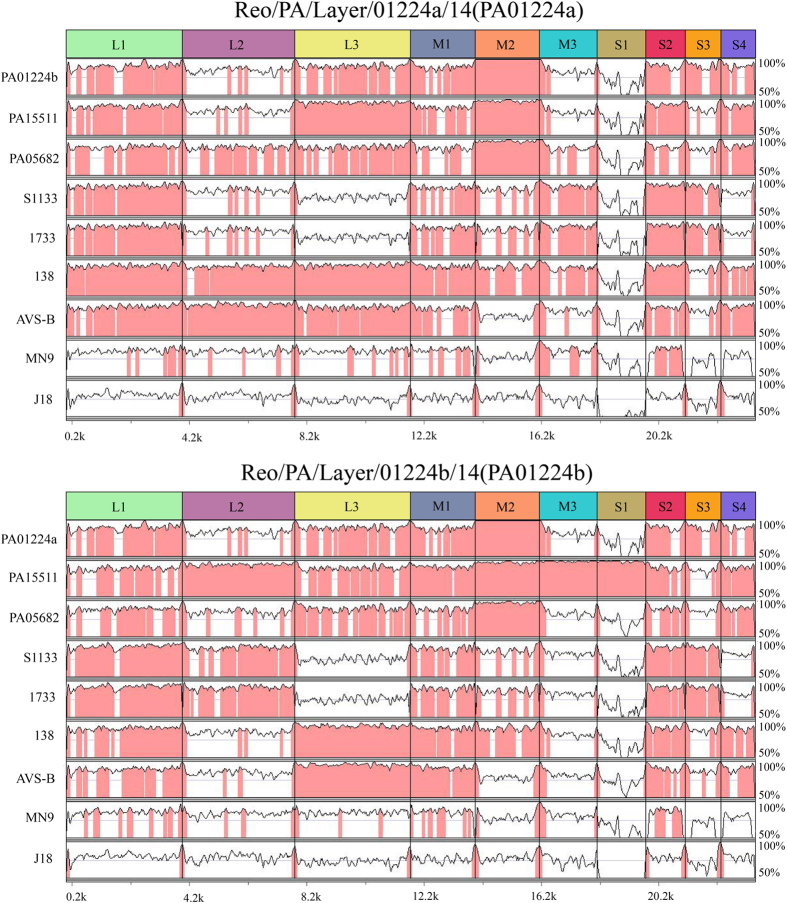
The mVISTA method for whole-genome nucleotide alignment. (**A**) Alignment results of the Reo/PA/Layer/01224a/14 variant strain in comparisons with the Reo/PA/Layer/01224b/14 variant strain and other 7 ARV reference strains (PA15511, PA05682, S1133, 1733, 138, AVS-B, MN9 and J18); (**B**) Alignment results of the Reo/PA/Layer/01224b/14 variant strain compared with the Reo/PA/Layer/01224a/14 variant strain and 7 other ARV reference strains (PA15511, PA05682, S1133, 1733, 138, AVS-B, MN9 and J18). The areas in pink represent ≥90% similarities, and the areas in white represent <90% similarities. The scale bar measures the approximate length of the concatenated genome.

The nt sequence similarity values of individual genome segments of the two co-infection variant strains, PA01224a and PA01224b, showed different divergence from each other and from all seven ARV reference strains (Fig. 4). The visualization of the genome in this way supported the phylogenetic results described above. High sequence similarities (>90%) were observed in most regions of L1, L3, M1, M2, S2, S3, and S4 segments between the two co-infection variant strains. In contrast, the two co-infection strains showed low identity (<85%) in the L2, L3, and S1 segments, with the lowest identity observed at the 3′ end of the S1 segment, corresponding to the σC-coding region. When compared with ARV reference strains, considerable genetic relatedness of the PA01224a variant and 138 strain was observed in 8 of the 10 genome segments, except for the most 3′ regions of the S1 segment and the most 5′ regions of the S3 segment. However, the highest identities of the S1 and S3 segments were observed between the PA01224a variant and the S1133 strain, with more than 57% and 88% nt similarities, respectively. The PA01224b variant strain showed close genetic relatedness with PA15511 strain of whole genome but more divergence from other reference strains compared with the PA01224a variant. The concatenated genome segments revealed that at least 6 reference strains (PA15511, PA05682, 1133, 1733 and AVS-B) shared high sequence similarity with the PA01224b variant in some genome segments. In addition, the M2 and S1 segments of the PA01224a and PA01224b variants, encoding the outer capsid proteins (μB and σC) of ARV, exhibited marked differences compared with vaccine strain S1133, indicating classic vaccine protection failure against the two co-infection variant strains. The duck-origin ARV strain-J18 shared low sequence identity with the two co-infection variant strains (<78.2%) throughout the entire genome, indicating no segment reassortment between the waterfowl-origin ARV and the two co-infection variant strains.

## Discussion

ARV-affected viral arthritis/tenosynovitis syndrome in domestic poultry was observed and described as a highly pathogenic and contagious poultry disease as early as over half a century ago in the 1950 s^22^,^23^,^24^, and continued studies were performed in 1960–70 s^25^,^26^,^27^. With the rapid development of the modern industrialized poultry business during the last several decades, ARV-affected poultry not only suffer the classic symptoms of viral arthritis/tenosynovitis but also the newly observed runting-stunting syndrome (RSS)^28^, respiratory disease^29^, enteric disease^30^, immunosuppression^31^, and malabsorption syndrome^32^. ARV-affected cases or flocks of broilers, turkeys, and layer chickens have been increasingly diagnosed in PA poultry in the US since 2011 until the present. Recent studies have revealed that a majority of the ARV outbreaks resulted from various variant strains of newly emerging ARVs^21^,^34^. In addition, highly pathogenic ARV variants also emerged in chickens^33^,^34^ and turkeys^35^ in other countries. Most of these emerging variants showed features of genome segment reassortment between historical ARV strains and high genetic diversity in the σC genes^36^.

The two co-infection variant strains described in the present study are the first report of two ARV variant strain co-infections naturally occurring in commercial young layer chickens, providing scientific evidence of the essential preconditions or requirements of potential reassortment between different ARVs^37^. It is difficult to detect and characterize the complete genomes of two co-infection variant strains without the most advanced NGS technology. In 2000, RT-PCR combined with Sanger sequencing was considered a powerful tool for the identification of ARV genotypes or pathotypes^20^, and this technique combined with cloning improvement was successfully used for the detection of two σC genes of the two co-infection variant strains described in the present study. However, direct Sanger sequencing for the RT-PCR product detected only one σC gene (PA01224a) in our initial test. The signal-to-noise sequencing ratio was appropriate, indicating that the RT-PCR product was a single component^38^. The plaque assay was also preferred for ARV co-infection characterizations in experimental co-infections with two ARVs in chicken fibroblasts^15^. The distinct viral growth kinetics in cell cultures or different viral titers in a test sample co-infected with ARVs would reduce the chances of the separation of co-infection strains.

Currently, the NGS technologies have revolutionized the field of genomics. In clinical virology, NGS is a highly efficient, fast processing technique, producing enormous amounts of information at low cost in a relatively short period of time^39^. The features of NGS in genome deep sequencing strategies could be used for the discovery of newly emerging viruses, the characterization of viral genome variability, and the identification and characterization of multiple co-infection viruses. NGS using the Illumina MiSeq platform was conducted with total RNA extracted from ARV-positive LMH cell cultures in the present study. The use of NGS in metagenomics studies facilitates the exploration of all ARV sequences present in the total RNA sample^40^, avoids the time-consuming isolation and culture of all co-infection strains individually, and requires no pre-information of the co-infecting ARVs. We performed the *de novo* assembly of the clean reads from NGS raw data and identified 19 ARV-related contigs in the test sample, corresponding to 10 genome segments of ARV. The nt BLAST results of all of the ARV contigs confirmed that the 19 ARV contigs were derived from two ARV genomes, having the same M2 segment contig. NGS-based RNA-seq provides a tool to measure and to compare gene transcription patterns at unprecedented resolution^41^. The NGS sequencing coverage was positively correlated with the amount of viral RNA, associated with the numbers of the viruses or the viral characteristic of growth kinetics in cell culture^15^. Therefore, the 19 ARV contigs were successfully separated into two viral genomes using a ‘maxcounts’ approach^42^. In S1 segments, the direct Sanger sequencing results indicated only one ARV variant (PA01224a) sequence in the co-infection sample. The failure to sequence the other ARV variant (PA01224b) σC gene might reflect a dramatic difference in the sequencing coverage between the two co-infection variant strains in their S1 segments (271.28x for S1a of PA01224a and 44.63x for S1b PA01224b) (Table 1), consistent with the preferential PCR amplification theory^43^.

The genomic analysis of the two co-infection variant strains, PA01224a and PA01224b, revealed that in addition to the same M2 segment, the nt and aa sequence identities between the two co-infection strains were moderate (nt: <88.8%; aa: <97.8%). Sequence comparison of the two co-infection strains with reference ARV strains confirmed that most genome segments of the co-infection strains shared high sequence identity with the reference strains, indicating that reassortment between the historical ARVs might directly result in the emergence of PA01224a and PA01224b variant strains. The S1 segment-encoded σC, the most variable protein in ARV, plays an important role for virus attachment^44^ and acts as an apoptosis inducer^45^. The results of the present study indicated that the sequence identities between the two co-infection strains were markedly low in the σC gene (nt: 58.1%; aa: 51.4%), and the identities were also low compared with the reference strains (nt:<65.3%; aa:<60.2%). The different sequencing coverage of the two co-infection strains resulting from growth kinetics of the virus might reflect the σC protein-based properties of the viruses in viral attachment and cell penetration^15^. In contrast with the high diversity of the σC gene, both PA01224a and PA01224b shared 100% identity in the M2 segment. In ARV, the M2 genome segment encodes the major μ-class outer capsid protein (μB) of the virus, involved in virus entry and transcriptase activation^46^. The specific M2 genome segment is required for the efficient establishment of a productive ARV infection in some host cells^47^, suggesting that the same M2 segment of the two co-infection strains might be associated with ARV entry and/or un-coating in the same layer chicken host cells.

Phylogenetic analysis of the individual segments or genes of the two co-infection strains revealed that both strains distantly evolved from each other in most L and M genomic segments, whereas these strains showed close relationships in σA, σB and σNS genes. The two co-infection strains and all reference strains formed host-associated groups in most genome segments, except M2 and σ-C. The M2 segment phylogenetic analysis revealed that the two co-infection stains of PA01224a and PA01224b clustered together with two other PA broiler ARV variant strains (PA05682 and PA15511) and the reference strain ARV 138, which distantly evolved from the chicken ARV vaccine strains, turkey strains, and waterfowl strains, suggesting that strain 138 might be the origin of the M2 segments of epidemic chicken ARV in PA. The phylogeny of the σC genes confirmed 5 genotyping clusters with high sequence identities (nt: >75%; aa: >85%) within the cluster and low sequence identities between each cluster (nt: <60%; aa: <65%). Distantly related with PA01224a in cluster 3, strain PA01224b fell into cluster 5 and showed a close phylogenetic relationship with the variant PA15511 strain, characterized as a novel arthritis ARV in broiler chicken in previous studies^21^, suggesting the bidirectional transmission of certain ARV strains in layer and broiler chickens. The highly pathogenic ARV strains showing this feature not only place the risk of vertical transmission form breeders to progenies but also increase the chances of ARV reassortment among different chicken hosts.

In conclusion, we identified two naturally occurring ARV variant strain co-infections in layer chickens using NGS. The two co-infection variants had the same M2 segment but were distantly evolved in nine other segments. The sequence comparison and phylogenetic analyses revealed that the genetic reassortment of the L, M and S segments between historical ARV strains could have led to the emergence of the two co-infecting ARV variants. The sequencing data analysis also predicted important roles for the μB protein in specific ARV host selection and the σC protein in viral growth kinetics. Thus, the findings of the present study have confirmed the actual existence of natural ARV co-infections and generated complete genomic data to obtain a better understanding the evolutionary relationship between ARV co-infection variants and reference strains.

## Methods

### Ethics statement

Tissue collections were conducted in accordance with procedure guidelines approved by the United States Department of Agriculture (USDA) (http://www.aphis.usda.gov/animal_health/lab_info_services/downloads/NecropsyGuideline.pdf). All animal procedures were performed in accordance with the regulations of The Pennsylvania State University (PSU) animal welfare and ethics guidelines (http://www.research.psu.edu/training/sari/teaching-support/animal-welfare-1) and approved by PSU Institutional Animal Care and Use Committees (IACUC). The virus propagation and test were carried out in our avian virology lab and all experimental protocols were approved by PSU Institutional Biosafety Committee.

### ARV isolate

The ARV isolate used in the present study was obtained from a routine diagnostic case of a submitted tendon pool sample from arthritic young layer chickens at 14 weeks of age. The ARV isolation was propagated in LMH cell cultures and was confirmed positive for ARV through a fluorescent antibody (FA) test using an ARV-conjugated antibody (ID No. 680 VDL 9501, NVSL, Ames, IA, USA) as described in our previous report^19^.

### RT-PCR and Sanger sequencing

Viral RNA was extracted from the ARV isolate in LMH cell culture fluid using an RNeasy Mini Kit (Cat. No. Z74106, QIAGEN, Valencia, CA, USA) according to the manufacturer’s instructions. The RT-PCR reaction was performed using a One Step RT-PCR Kit (Cat. No. 210212, QIAGEN, Valencia, CA, USA) with two degenerate primers P1 and P4, corresponding to the σC gene of ARV^20^. The RT-PCR products were obtained through 1% agarose gel electrophoresis and were purified using a gel extraction kit (Cat. No. 04113KE1, Axygen, Tewksbury, MA, USA) according to the manufacturer’s instructions. Purified RT-PCR product was cloned into pGEM-T easy vector using pGEM-T easy Vector system (Promega, Madison, WI, USA), and then transformed into competent *Escherichia coli* JM109 cells (Promega, Madison, WI, USA) in accordance with the manufacturer’s instructions. The recombinant plasmids from positive colonies were isolated by using E.Z.N.A.^®^ Plasmid DNA Mini Kit I (Omega Bio-tek, Norcross, GA, USA) per the manufacturer’s instructions. The concentration of the purified RT-PCR product and recombinant plasmids were obtained using a NanoDrop™1000 (Thermo Scientific, Waltham, MA, USA) spectrophotometer and were subsequently submitted to the Penn State Genomics Core Facility for Sanger sequencing.

### Next-generation sequencing

RNA libraries were constructed from total RNA samples using the TruSeq Stranded Total RNA Sample Prep Kit (Cat. No. RS-122–2201, Illumina, San Diego, CA, USA) according the manufacturer’s protocol, but without the initial poly-A enrichment step. The library size and quality was assessed using an Agilent Bioanalyzer (Agilent Technologies, Santa Clara, CA, USA). The library concentration was assessed through qPCR using the KAPA Library Quantification Kit Illumina Platforms (Cat. No. KR0405, Kapa Biosystems, Wilmington, MA, USA). After quality testing, the RNA libraries were directly sequenced on an Illumina MiSeq using 150-nt single-read sequencing according to the manufacturer’s protocol.

### Viral genome assembly

The pipeline for the NGS raw reads analysis has been described in a previous study^9^. Briefly, all generated reads were trimmed to remove sequencing adaptors and low-quality reads prior to further analysis. After the reads mapping to chicken, bacteria, or rRNA were removed using the sortMeRNA^48^ and BWA-MEM programs^49^, the remaining reads were processed using de novo SPAdes assembly software (ver 3.5.0)^50^ to obtain assembled contiguous sequences (contigs). The ARV-related contigs were extracted after alignment to the reference genome using LASTZ software^51^, and subsequently, the sequences were mapped back using all raw reads for contigs improvement. Moreover, the consensus sequences from the re-mapping reads and LASTZ contig alignment were obtained using SAMtools commands^52^.

### Sequence analyses

The ARV ORF predictions, amino acid (aa) translations, and sequence alignments were performed using the Lasergene 12 Core Suite (DNASTAR, Inc. Madison, WI, USA). The assembled ARV contigs were submitted to BLASTN online searching in GenBank (http://blast.ncbi.nlm.nih.gov/Blast.cgi) to obtain the reference sequence with the most homology. Phylogenetic trees of ten ARV segments (genes) were generated using the neighbor-joining method and were validated through bootstrap analysis with 1000 replications in the MEGA 5.0 program. Sanger sequencing, NGS sequencing coverage, NGS assembled contigs, viral ORFs, intra-host single-nucleotide variants (iSNVs) and nt differences between the study and reference strains were visualized through the Circos software package^53^. The analysis and visualization of whole genome alignments were performed using the mVISTA online platform (http://genome.lbl.gov/vista/mvista/submit.shtml). The sequencing coverage of each assembled contig was calculated using CLC Genomics Workbench V7.5 software (QIAGEN, Boston, MA, USA) and was separated into two strains based on average coverage. Statistically significant differences between the two separated strains were determined using the two-tailed Student’s t-test in the Microsoft Excel program. The GenBank accession numbers for ARV reference strains (PA15511, PA05682, S1133, 1733, 138, AVS-B, MN9 and J18) are listed in Supplementary Table S1.

## Additional Information

**How to cite this article**: Tang, Y. *et al.* Detection and characterization of two co-infection variant strains of avian orthoreovirus (ARV) in young layer chickens using next-generation sequencing (NGS). *Sci. Rep.* **6**, 24519; doi: 10.1038/srep24519 (2016).

## Supplementary Material

Supplementary Information

## Figures and Tables

**Table 1:** De novo assembly contigs of two co-infection variant strains (Reo/PA/Layer/01224a/14 and Reo/PA/Layer/01224b/14) of avian orthoreovirus (ARV) isolated from ARV-affected young layer chickens

Segment length (bp)	Contig name	Highest similarity to the reference ARV strain in GenBank	Identities (%)	SNVs	Mapped reads	Average coverage
3958	L1a	138 strain segment L1 lambda A gene (EU707933)	91	13	3936	125.82
3958	L1b	2408 strain segment L1 lambda A gene (AY641742)	91	4	1746	59.06
3829	L2a	AVS-B strain segment L2 lambda B gene (FR694192)	93	0	3106	106.79
3829	L2b	1733 strain segment L2 lambda B gene (KF741707)	90	0	1341	46.04
3907	L3a	AVS-B strain segment L3 lambda C gene (FR694193)	95	0	914	30.72
3907	L3b	138 strain segment L3 lambda C gene (EU707937)	92	1	5978	202.55
2283	M1a	138 strain segment M1 muA gene (AY557188)	91	0	3053	184.38
2283	M1b	AVS-B strain segment M1 muA gene (FR694194)	91	1	1168	65.44
2158	M2	Reo/PA/Broiler/05682/12 segment M2 muB gene (KM877329)	97	0	4256	254.01
1996	M3a	1017-1 strain segment M2 muNS gene (AY573905)	89	0	405	26.82
1996	M3b	S1133 strain segment M2 muNS gene (KF741761)	90	0	3069	204
1644	S1a	T1781 segment S1 sigma C genes (KC865792)	82	0	3560	271.28
1644	S1b	Reo/PA/Broiler/15511/13 segment S1 sigma C genes (KP731617)	98	0	564	44.63
1324	S2a	S1133 strain segment S2 sigma A gene (KF741763)	92	1	1759	174.96
1324	S2b	526 strain segment S2 sigma A gene (KF741703)	91	0	636	65.05
1202	S3a	1733 strain segment S3 sigma B gene (KF741714)	92	1	1178	134.3
1202	S3b	526 strain segment S3 sigma B gene (KF741704)	94	1	2175	246.3
1192	S4a	AVS-B strain segment S4 sigma NS gene (FR694200)	91	1	404	46.89
1192	S4b	526 strain segment S4 sigma NS gene (KF741705)	93	1	2138	247.95

**Table 2:** Genome features of two co-infection variant strains (Reo/PA/Layer/01224a/14 and Reo/PA/Layer/01224b/14) of avian orthoreovirus (ARV) isolated from ARV-affected young layer chickens.

Reo/PA/Layer/01224a/14						Reo/PA/Layer/01224b/14					
Contig name	Average coverage	Encoded protein	Length (bp)			Contig name	Average coverage	Encoded protein	Length (bp)		
			5′end	ORF	3′end				5′end	ORF	3′end
L1a	125.82	λA	20	3882	56	L1B	59.06	λA	20	3882	56
L2a	106.79	λB	13	3780	36	L2B	46.04	λB	13	3780	36
L3b	202.55	λC	12	3858	37	L3A	30.72	λC	12	3858	37
M1a	184.38	μA	12	2199	72	M1B	65.44	μA	12	2199	72
M21	254.01	μB	29	2031	98	M2a	254.01	μB	29	2031	98
M3b	204	μNS	24	1908	64	M3A	26.82	μNS	24	1908	64
S1a	271.28	p10	22	300	33	S1B	44.63	p10	22	300	33
		p17		459				p17		441	
		σC		981				σC		981	
S2a	247.95	σA	15	1251	58	S2B	46.89	σA	15	1251	58
S3b	246.3	σB	30	1104	68	S3A	134.3	σB	30	1104	68
S4b	174.96	σNS	23	1104	65	S4A	65.05	σNS	23	1104	65

The two avian orthoreovirus (ARV) variant strains had the same M2 contig.

**Table 3:** Comparison of segment 5′ and 3′ non-coding regions of two co-infection variant strains (Reo/PA/Layer/01224a/14 and Reo/PA/Layer/01224b/14) of avian orthoreovirus (ARV) with ARV reference strains.

ARV strain	Host	Terminal region sequences (5′ to 3′)
Reo/PA/Layer/01224a/14	Layer Chicken	GCUUUU^U^/_C_…UA^U^/_C_UCAUC
Reo/PA/Layer/01224b/14	Layer Chicken	GCUUUU^U^/_C_…UA^U^/_C_UCAUC
Reo/PA/Broiler/05682/12	Broiler Chicken	GCUUUU^U^/_C_…UA^U^/_C_UCAUC
Reo/PA/Broiler/15511/13	Broiler Chicken	GCUUUU^U^/_C_…UA^U^/_C_UCAUC
S1133	Broiler Chicken	GCUUUUU…UA^U^/_C_UCAUC
1733	Broiler Chicken	GCUUUU^U^/_C_…UA^U^/_C_UCAUC
138	Broiler Chicken	GCUUUU^U^/_C_…UA^U^/_C_UCAUC
AVS-B	Broiler Chicken	GCUUUU^U^/_C_…UA^U^/_C_UCAUC
2408	Broiler Chicken	GCUUUUU… UAUUCAUC
MN9	Turkey	GCUUUUU… UAUUCAUC
J18	Muscovy Duck	GCUUUUU…UA^U^/_C_UCAUC
D20/99	Goose	GCUUUU^U^/_C_…UA^U^/_C_UCAUC

**Table 4:**
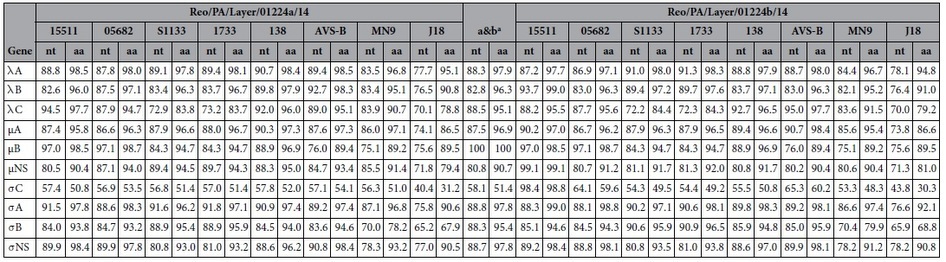
Sequence identities of genome segments and proteins between the two co-infection variant strains (Reo/PA/Layer/01224a/14 and Reo/PA/Layer/01224b/14) and reference strains of avian orthoreovirus (ARV).

1 Genome sequence identities between two PA ARV field co-infection strains.
