# Supplementary material for: Detection and characterization of two co-infection variant strains of avian orthoreovirus (ARV) in young layer chickens using next-generation sequencing (NGS)
Source: Sci Rep. 2016 Apr 19;6:24519. doi: 10.1038/srep24519 (PMC4835796; doi:10.1038/srep24519)
Supplement: Supplementary Information [file srep24519-s1.pdf]

Detection and characterization of two co-infection variant strains of avian orthoreovirus (ARV) in young layer chickens using next-generation sequencing (NGS)

Yi Tang[1], Lin Lin[1], Aswathy Sebastian[2], Huaguang Lu[1]

Supplementary Table S1. GenBank accession numbers for the complete genome segments of eight avian orthoreovirus (ARV) reference strains (PA15511=Reo/PA/Broiler/15511/13, PA05682=Reo/PA/broiler/05682/12, AVS-B, S1133, 138, 1733, 176, MN9 and J18).

Segment	PA15511	PA05682	AVS-B	S1133	138	1733	MN9	J18
**L1**	KP731611	KM877325	FR694191	KF741756	EU707933	KF741706	KJ865905	JX478260
**L2**	KP731612	KM877326	FR694192	KF741757	EU707935	KF741707	KJ865895	JX478261
**L3**	KP731613	KM877327	FR694193	KF741758	EU707937	KF741708	KJ865885	JX478262
**M1**	KP731614	KM877328	FR694194	KF741759	AY557188	KF741709	KJ874314	JX478263
**M2**	KP731615	KM877329	FR694195	KF741760	AY557189	KF741710	KJ874291	JX478264
**M3**	KP731616	KM877330	FR694196	KF741761	AY557190	KF741711	KJ874268	JX478265
**S1**	KP731617	KM877331	FR694197	KF741762	AF218359	KF741712	KF872241	JX478266
**S2**	KP731618	KM877332	FR694198	KF741763	AF059717	KF741713	KF872253	JX478267
**S3**	KP731619	KM877333	FR694199	KF741764	AF059721	KF741714	KF872261	JX478268
**S4**	KP731620	KM877334	FR694200	KF741765	AF059725	KF741715	KF872277	JX478269
